# Socioculturally Appropriate Internet-Based Geriatric Care Model for Older Adults Living With HIV: Experience-Based Co-Design Approach

**DOI:** 10.2196/67122

**Published:** 2025-05-27

**Authors:** Kristina M Kokorelias, Marina B Wasilewski, Dean Valentine, Andrew D Eaton, Erica Dove, Paige Brown, Stuart McKinlay, Christine L Sheppard, Esther Su, Hardeep K Singh, Ashley Flanagan, Alice Zhabokritsky, Reham Abdelhalim, Rabea Parpia, Rahel Zewude, Laura Jamieson, Sharon Walmsley, Luxey Sirisegaram

**Affiliations:** 1 Section of Geriatric Medicine Department of Medicine Sinai Health System and University Health Network Toronto, ON Canada; 2 Department of Occupational Science and Occupational Therapy University of Toronto Toronto, ON Canada; 3 Rehabilitation Sciences Institute University of Toronto Toronto, ON Canada; 4 National Institute on Ageing Toronto Metropolitan University Toronto, ON Canada; 5 St John’s Rehab Research Program Sunnybrook Health Sciences Centre Toronto, ON Canada; 6 Faculty of Social Work Saskatoon Campus University of Regina Saskatoon, SK Canada; 7 Factor-Inwentash School of Social Work University of Toronto Toronto, ON Canada; 8 Undergraduate Medical Education Temerty Faculty of Medicine University of Toronto Toronto, ON Canada; 9 KITE Toronto Rehabilitation Institute University Health Network Toronto, ON Canada; 10 Infectious Diseases Department of Medicine University Health Network Toronto, ON Canada; 11 Division of Infectious Diseases Department of Medicine University of Toronto Toronto, ON Canada; 12 CIHR Canadian HIV Trials Network Vancouver, BC Canada; 13 Burlington Ontario Health Team Joseph Brant Memorial Hospital Burlington, ON Canada; 14 St Michael’s Hospital Toronto, ON Canada; 15 Ontario Federation of Indigenous Friendship Centres Toronto, ON Canada

**Keywords:** older adults, HIV, virtual care, co-design, qualitative research

## Abstract

**Background:**

Older adults living with HIV face challenges accessing regular geriatric care, and while virtual care services could offer a solution, they may come with limitations.

**Objective:**

This study aimed to co-design a culturally appropriate virtual care model tailored to older adults’ needs using the experience-based co-design methodology.

**Methods:**

We used a qualitative, experience-based co-design approach with 19 older adults living with HIV. The process involved 3 phases: identifying needs through interviews and questionnaires, codeveloping a care model prototype through focus groups and a workshop, and refining the model using feedback from a world café format. Data were analyzed using thematic content analysis.

**Results:**

The co-design process led to a virtual care model prototype that directly addressed participants’ key needs. These included personalized communication methods, simplified technology interfaces for easier access, and culturally responsive care practices. Participants emphasized the importance of privacy in virtual consultations, flexible scheduling to accommodate health fluctuations, and ongoing support for managing both HIV and aging-related conditions. Their feedback shaped a model designed to bridge service gaps, offering a more inclusive, accessible, and patient-centered approach to virtual geriatric care.

**Conclusions:**

This study co-designed a potential virtual geriatric care model grounded in the experiences of older adults living with HIV. By integrating participants’ insights throughout the design process, the model offers a promising approach to improving care for this vulnerable population. Future directions for research to test this model are proposed.

## Introduction

### Background

The global population is aging, with the number of people aged ≥50 years projected to double by 2050, reaching approximately 2 billion individuals worldwide [1]. The aging global population also includes a growing number of older adults living with HIV [2]. Due to advancements in combination antiretroviral therapy, the life expectancy of people living with HIV has significantly increased [3], leading to the first generation of people living with HIV now reaching geriatric age [4]. This demographic shift brings unique challenges as older adults living with HIV experience a complex interplay of age-related comorbidities and HIV-related health issues [5-7].

Older adults living with HIV encounter a myriad of challenges that significantly impact their geriatric care. Medically, these individuals are more likely to experience comorbid conditions, including cardiovascular disease [6,8,9], diabetes [6,10], and osteoporosis [11-13], which can complicate HIV management and treatment [14]. These comorbidities complicate treatment regimens and require coordinated care, which may not always be available [15,16]. The specialization of health care often leads to fragmented care, where health care providers focus solely on HIV management without considering the broader geriatric care needs of the patient, such as mobility, nutrition, and mental health [17,18]. Furthermore, the physiological changes associated with aging, combined with the long-term effects of HIV and its treatment, can exacerbate these comorbidities, leading to a higher burden of illness and increased health care use [19]. Psychologically, older adults living with HIV may experience higher rates of depression [20-22], anxiety [23,24], and cognitive impairment [25-27] than their younger counterparts living with HIV. The stigma associated with both aging and HIV can combine and contribute to social isolation, discrimination in health care settings, and declines in mental health and overall well-being [28-30]. In addition, older adults may encounter ageism within health care settings, which can negatively affect the quality of care they receive and their willingness to seek further treatment [31-33]. Thus, the increase in older people living with HIV underscores the increasing need for comprehensive geriatric care that addresses the complex health needs of older adults [34,35].

HIV is highly stigmatized, and this can exacerbate other experiences of stigma and discrimination [32,36,37]. Notably, geriatric care (ie, care delivered specifically for older adults) is most effective when it meets the sociocultural needs of its patients [38,39]. Socioculturally appropriate care refers to health care that respects and responds to the cultural and social needs of patients, integrating their backgrounds, beliefs, and values into their care plans [40-43]. This approach is crucial for improving patient outcomes, enhancing satisfaction with care, and reducing health disparities [44-46]. However, delivering socioculturally appropriate care, particularly in the intersection of geriatrics and HIV, presents unique challenges [43]. These include a lack of cultural competency training [47,48], insufficient resources [49-51], language barriers [52,53], and implicit biases among health care providers [54-56], which can lead to misunderstandings and suboptimal care [57]. Moreover, individuals aging with HIV may come from diverse countries and have varying sexual identities and unique histories of trauma, discrimination, and stigma that differ from those of the general population [36,37,58]. Cultural mismatches such as failing to address the stigma associated with HIV in certain cultures can result in reduced patient engagement, lower treatment adherence, and worse health outcomes [36,59]. Therefore, addressing these challenges is essential to provide effective geriatric care to diverse people living with HIV.

Virtual care holds significant potential to address some of the challenges faced by older people living with HIV [60-63]. For instance, virtual care can help facilitate regular check-ins and monitoring without the need for frequent in-person clinic visits, which is particularly beneficial for those with mobility issues or transportation obstacles or for those living in remote areas [64-66]. As one example, virtual interventions for rural older veterans living with HIV have been shown to improve access to care and patient health outcomes, including quality of life, medication adherence, depressive symptoms, internalized stigma, and loneliness [67]. Virtual platforms can also provide a discrete way for patients to access care, potentially reducing stigma-related barriers to seeking health care in person [68-70].

While virtual care has revolutionized health care delivery, it also presents several challenges that need to be addressed for effective implementation [71,72]. One major issue is the digital divide, where limited access to technology and reliable internet connectivity, especially in rural and underserved areas, hampers the ability of patients to engage in virtual care [73-75]. In addition, digital literacy poses a significant barrier as many patients, particularly older adults, may lack the knowledge and skills to navigate digital health platforms effectively [17,73,76]. There is a need to develop specialized socioculturally appropriate virtual geriatric care models tailored to the unique needs of diverse older people, including those living with HIV. Geriatric care refers to the specialized medical and supportive care tailored to meet the complex health and social needs of older adults, encompassing chronic disease management, functional support, and holistic well-being [50].

Delivering geriatric care virtually for people living with HIV presents both opportunities and significant challenges. While virtual care can enhance access to health care for older adults living with HIV, particularly in underserved or rural areas, it also requires careful consideration of various factors. One of the primary challenges is the complexity of managing multimorbidity, a hallmark of aging in people living with HIV, which often involves the treatment of multiple chronic conditions, including cardiovascular disease, diabetes, and mental health disorders [77,78]. Virtual care platforms may struggle to capture the full range of physical and cognitive assessments needed to effectively manage these conditions. In addition, older adults with HIV may face difficulties with digital literacy, which can hinder their ability to use virtual care tools effectively [79]. This is particularly true for older adults who may already experience cognitive decline or sensory impairments. The quality of the therapeutic relationship can also be affected in virtual settings as the absence of in-person interactions may reduce the ability of health care providers to observe subtle nonverbal cues and create a trusting environment [79]. Moreover, people living with HIV may have unique psychosocial challenges, such as stigma and social isolation, which can be exacerbated by virtual care [79]. Addressing these challenges requires the development of specialized, culturally competent virtual care models that incorporate both technological solutions and social support structures to ensure holistic, patient-centered care for older adults living with HIV [43].

Co-design is a valuable approach in developing and refining health interventions and services as it actively involves key players in the design process to ensure that the resulting solutions are highly relevant and effective for end users [80]. By engaging those who will ultimately benefit from these interventions, co-design fosters a deeper understanding of their needs, preferences, and sociocultural contexts, leading to more tailored and impactful outcomes [80,81]. By involving older adults in the design process, the resulting solutions will be more relevant and effective in addressing their unique health challenges and sociocultural contexts. *Co-design* promotes cultural sensitivity by incorporating the perspectives and experiences of diverse patient populations into the design and delivery of health services [82]. This is particularly important for older people living with HIV, who may face additional stigma and barriers underpinned by the intersection of sociocultural considerations and demographic factors such as age [83]. Addressing the gaps in the literature will ensure that the unique health challenges and sociocultural contexts of older people living with HIV are considered, leading to more effective and inclusive geriatric-HIV health care interventions [43].

### Objectives

This paper reports on the co-design of a culturally appropriate virtual care model for older adults living with HIV using an experience-based co-design (EBCD) approach [84]. By involving older people living with HIV, this study sought to create a person-centered care model that enhances their health care experience and outcomes [85].

## Methods

### Study Design

In this community-based participatory research (CBPR), we used a qualitative EBCD methodology [84] that integrated participants as co-designers (details can be found in a published protocol [43]). CBPR is an approach that emphasizes collaboration between researchers and community members throughout the research process [86,87]. It aims to address community-identified needs and promote social change by integrating knowledge and action for social justice [86,88]. Co-design involves meaningful engagement of end users in the research design process, encompassing participation at every stage of the research and varying in intensity from passive involvement to highly active participation [84,89-91]. To achieve our research objective of co-designing a geriatric-HIV virtual care model, we adapted and used a variety of participatory EBCD methods, including interviews, focus groups, scenario design, and world cafés [84,92]. We briefly describe our methods in the following sections.

### Ethical Considerations

Ethical considerations in CBPR include ensuring informed consent, where community members fully understand the research process and its implications [93]. This study was approved by the Sinai Health Research Ethics Board (approval 23-0106-E). All participants provided written informed consent to take part in the study, be audio recorded, and have anonymous quotations published. The participants were given the opportunity to have access to a translator and ask to clarify any concerns before signing the consent form. Participants in the focus groups and workshop were provided with refreshments and lunch and were reimbursed for their travel expenses. Participants in all phases also received an honorarium of $43 USD for their time, compensated at an hourly rate. Our study also emphasized community ownership of research findings, allowing the advisory committee to make decisions about how results are disseminated. All data shared with the advisory committee were anonymous.

### Researcher Positionality

The research team (n=18) consisted of Canadian experts in co-design methodologies with diverse backgrounds and expertise in HIV research, equity-informed health care, health service research, and implementation science. This interdisciplinary team included PhD-trained researchers and clinicians specializing in geriatrics, family medicine, and infectious diseases, as well as occupational therapists, social workers, health service administrators, and peer researchers with lived experience.

A community-based advisory team (n=10) was established before protocol development to ensure ongoing engagement throughout the project. This advisory team comprised knowledge users such as administrators from nonprofit organizations serving older persons living with HIV, including shelter staff, health care providers, charity organizations, and community centers. In addition, the advisory team included older individuals living with HIV and clinicians specializing in geriatric care. Regular individual meetings with principal investigators were conducted throughout the research process.

### Recruitment and Participants

Our study sample was distinct from our advisory committee and included older individuals who self-identified as HIV positive; were aged ≥50 years; and resided in Ontario, Canada. We purposively sought diversity across various dimensions: (1) sex and gender, (2) age, (3) ethnicity and race, (4) socioeconomic status, (5) previous use of virtual geriatric care (ie, yes or no), (6) geographical location (rural vs urban), (7) time living with HIV, (8) non-English first language, and (9) level of educational attainment.

We used quota and purposive sampling to ensure a representative sample [94,95]. Our advisory team engaged in community outreach at relevant meetings and events to assist with recruitment, leveraging their networks and organizations. We also posted study flyers at community-based organizations, religious institutions, and culturally oriented events. Finally, our advisory team’s websites and social media platforms were used to promote the study. All participants contacted the research coordinator to learn more about the study, and eligibility was confirmed based on the demographic characteristics to ensure alignment with our purposeful sampling technique.

### Data Collection

Data collection occurred in 3 phases, which are outlined in this section and published in a previous protocol [43]. No deviations were made, aside from the fact that no focus groups occurred in phase 1. Phase 1 focused on understanding participants’ needs and perspectives through semistructured interviews. The interviews were conducted by a trained research assistant with support from the primary author. The interviews focused on participants’ experiences throughout their illness trajectory, current and anticipated needs for geriatric care, and priorities for virtual care. To ensure effective purposive quota sampling, demographic information was collected from participants at the time of recruitment, allowing us to determine whether they met the specific criteria for inclusion in the study. This demographic questionnaire captured diverse lived experiences and socioeconomic data relevant to this study’s aims. Once participants were recruited, all interviews were audio recorded, professionally transcribed, and reviewed for accuracy by a research assistant. The narrative summaries of the interviews were created by the first author based on both the interviewer’s notes and recollections and the transcripts of the interviews. These summaries were developed to provide a comprehensive overview of each interview, highlighting key themes, insights, and participant perspectives. Following their creation, the narrative summaries were shared with the research team and the advisory team via email and team meetings. The narrative summaries were included in the dataset for analysis and were considered part of the field and meeting notes, providing a synthesized account of key themes and insights from the interviews to support the interpretation and development of findings.

Before initiating the co-design process, we prioritized trust building to create a comfortable and inclusive environment for participants. To minimize power imbalances, we limited the number of researchers present, ensuring that the space felt less formal and more participant driven. In addition, we asked participants whom they wanted involved in the process, allowing them to shape the composition of the sessions. These efforts fostered a sense of ownership, encouraged open dialogue, and reinforced the collaborative nature of the co-design approach.

Phase 2 involved 2 focus groups and a workshop, all drawing on the same participants to apply an EBCD approach to develop a prototype for a culturally appropriate HIV virtual care model. The focus groups primarily aimed to gather in-depth qualitative data through structured discussions that allowed participants to share their experiences and insights about virtual care. In contrast, the workshop was more interactive and aimed at synthesizing the findings from the focus groups, where participants worked collaboratively to co-design and refine the elements of the HIV virtual care model. In preparation for phase 2, participants in the focus groups were provided with a detailed presentation summarizing the main needs identified in phase 1 before the workshop formally began. This presentation, based on comprehensive data collected during the initial phase, highlighted key insights into the unique challenges faced by older adults living with HIV. By sharing this information with the participants, we informed them about critical areas to address in the design process, ensuring that their feedback in the focus groups was grounded in the context of the identified needs. The focus groups and workshop were jointly led by a trained neutral facilitator and peer researcher who was trained through a structured program designed to build both facilitation and research skills. The training included an overview of the research objectives, ethical considerations, and techniques for creating a supportive and nonjudgmental environment for participants. The peer researcher was also trained in active listening, group dynamics, and how to manage sensitive topics, particularly those related to the experiences of older adults living with HIV. The workshop, guided by a modified 4D cycle of Appreciative Inquiry [96], aimed to identify strengths in existing care models and design principles for improved virtual care, accommodating both in-person and virtual attendance [97]. The modified 4D cycle of Appreciative Inquiry involves the phases of *Discovery*, *Dream*, *Design*, and *Destiny*, focusing on identifying strengths, envisioning an ideal future, cocreating actionable plans, and implementing sustained changes [96]. The *Definition* phase was also included for greater adaptability to specific contexts and goals. Discussions used visual tools such as concept mapping and word clouds [98] that were kept for analysis. All verbal focus group data were audio recorded and professionally transcribed. Postworkshop reflections with the research and advisory teams guided phase 3.

In phase 3, there were 10 returning participants from phase 2 and 2 participants new to the study. The design principles for the culturally appropriate HIV virtual care model were developed using a world café format [99]. Small group discussions fostered dynamic exchanges, integrating feedback on proposed solutions and outlining recommendations for implementation and uptake. Facilitated by a peer researcher and supported by research team members, these sessions aimed to ensure participant engagement and generate robust data for ongoing project development [99]. Memos and reflexive notes were taken by the research team throughout the study. Transcripts and notes were anonymized by removing details that could lead to participant identification. No names of participants were used in the analysis or reporting of the results.

### Data Analysis

The dataset comprised audio recordings, transcripts, field and meeting notes, reflexive notes, and physical artifacts designed by participants. The analysis team, consisting of research team members and select advisory committee members, conducted thematic content analysis [100,101]. The field and meeting notes were reviewed as part of the data analysis process, contributing to the identification of key themes. In phase 1, data analysis occurred concurrently with data collection to determine thematic saturation [102]. Thematic saturation was established when no new codes or concepts emerged from the data, indicating that additional interviews were unlikely to yield novel insights. Transcripts were coded using the NVivo software (version 14; QSR International) by the principal investigator and the research assistant. A codebook developed through inductive and deductive processes informed by the knowledge-to-action framework was used and refined iteratively through team meetings [103,104]. Physical artifacts created by participants during the workshop, including Post-it notes, drawings, and summary sheets, were also systematically analyzed and integrated into the overall data analysis process to enrich and deepen the understanding of participants’ perspectives. Once scanned, the images were categorized based on themes and topics that emerged from the discussions. This allowed the research team to examine not only the content of the physical artifacts but also the ways in which participants visually expressed their ideas and emotions. The drawings and Post-it notes, for example, provided additional insights into participants’ cognitive and emotional responses, offering a layer of qualitative data that complemented the verbal responses collected during the sessions. The images were analyzed using a thematic approach, identifying recurring patterns, symbols, and motifs that reflected key aspects of participants’ experiences. Peer researchers were sent the preliminary findings 1 week ahead of the focus groups and workshop to enable thorough review and reflection before the subsequent phases. Themes were identified through comparative analysis and refined through team discussions, with a thematic map aiding in refining final themes to inform phase 2. Phase 2 and phase 3 continued with reflexive thematic content analysis to consolidate findings from the co-design workshop and refine themes for subsequent phases [101], including the world café discussions. The analysis refined the themes that emerged during these sessions and ensured that the feedback from the world café format was directly incorporated into the final design recommendations for the virtual care model. All researchers reviewed and endorsed the final version of the manuscript as coauthors to ensure the accuracy and completeness of the findings presented. A lay version of the manuscript was also sent to all members of the advisory team and participants of the study. Participants were invited to provide their feedback via email up to the point of manuscript submission.

### Validation and Reliability Strategies

Rigor and trustworthiness in this study were ensured through several methodological strategies. First, we used a qualitative EBCD methodology, integrating participants as co-designers throughout the research process, which facilitated meaningful end-user engagement and ensured that participant perspectives were central to the study’s design and outcomes. This approach was consistent with established principles of CBPR [86,105]. Second, we maintained methodological rigor by adhering strictly to our published protocol [43], which detailed the study procedures and ensured consistency in data collection and analysis methods. Our adherence to the protocol provided a structured framework for data collection and interpretation while remaining adaptable to participant and advisory committee input and emerging insights. This balance ensured that, while following a predefined approach, we also responded to and incorporated collaborators’ feedback, thus enhancing the relevance and responsiveness of the methodology. Third, the study design included diverse methods that enhanced the richness and depth of the data collected and contributed to the triangulation of data sources [106,107]. The analysis team, consisting of research team members and selected advisory committee members, engaged in iterative coding and theme development supported by regular team meetings and peer review processes. This collaborative approach enhanced the reliability and credibility of the findings by ensuring that interpretations were grounded in the data and reflective of diverse perspectives [108]. Finally, transparency and reflexivity were maintained throughout the research process. That is, reflexive notes, memos, and detailed documentation of methodological decisions were kept, providing transparency in data interpretation and analysis. Regular interactions with the advisory team and participants, including sharing preliminary findings and seeking feedback, further strengthened the trustworthiness of this study by ensuring that the findings resonated with participant experiences and priorities [108].

## Results

### Overview

**Figure 1:**
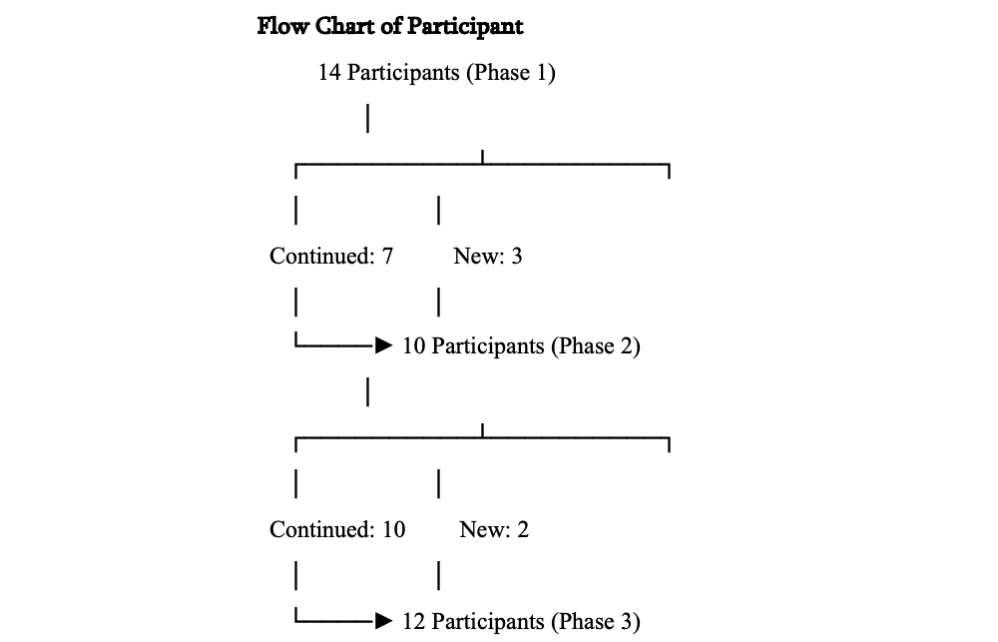
Flowchart of participants.

The research team conducted the co-design process between June 2023 and June 2024. In total, 19 unique participants took part in this study, with 14 (74%) participants in phase 1, a total of 10 (53%) participants in phase 2 (n=7, 70% from phase 1), and 12 (63%) participants in phase 3 (n=10, 83% from phases 1 and 2). Multimedia Appendix 1 outlines the participant characteristics, and Figure 1 shows a flowchart of the participants.

Participants co-designed a culturally appropriate and flexible HIV virtual care model that was rooted in empathy and addressed both the clinical, psychological, and social elements and the emotional aspects of aging with HIV. Phase 1 aimed to understand participants’ needs regarding virtual care, revealing four key findings: (1) the desire for accessible and integrated health care; (2) the complications caused by sociocultural and demographic factors; (3) the benefits of peer support without replacing formal care; and (4) the potential of virtual care to address some barriers, although with caveats related to access and technology. Participants expressed a strong need for easily accessible, coordinated care, particularly for older adults living with HIV who face complex, overlapping health and social challenges. They highlighted the difficulties of navigating the health care system, noting that virtual care could reduce barriers but only if it accounts for digital literacy and technology access. In addition, while peer support was valued, participants cautioned against overreliance on it, emphasizing that virtual care should supplement, not replace, formal services. They also pointed out that virtual care could improve access, especially for individuals facing logistical barriers, but it must be designed with flexibility and continuity in mind. In phase 2, the focus shifted to ideating solutions for the virtual geriatric-HIV care model. Participants suggested a multimodal care approach encompassing various forms of care delivery (eg, in-person and virtual visits and digital health records). They also stressed the importance of education and clear communication regarding technology use, ensuring that older adults are informed and confident in using virtual platforms. Privacy and data security were significant concerns, with participants requesting transparency about who accesses their information and how it is used. In addition, participants emphasized the need for reminder systems to help maintain continuity of care and reduce missed appointments. These solutions were aimed at improving both the practicality and trustworthiness of virtual care for older adults living with HIV. Phase 3 focused on co-designing actionable plans, with participants emphasizing the importance of linguistic and cultural equity in virtual care. They stressed that care models should be inclusive of diverse populations, particularly those who face language barriers. Participants highlighted the need for culturally appropriate services to ensure equitable care, pointing out that older adults from different cultural backgrounds often experience compounded difficulties when accessing health care services. The plans developed during this phase aimed to refine and prioritize these solutions to create a more inclusive and effective virtual care model for older adults living with HIV. Throughout the following sections, we illustrate the findings using quotes labeled with participant ID, race or ethnicity, gender, and age.

### Phase 1: Understanding Participants’ Needs Regarding Virtual Care

We identified the following key and interrelated insights: (1) older adults living with HIV want accessible and integrated care, (2) intersecting sociocultural and demographic factors complicate receipt of care for older adults living with HIV, (3) peer support is beneficial but should not replace formal health care services and supports, and (4) virtual care can address barriers to care with access caveats.

#### Older Adults Living With HIV Want Accessible and Integrated Care

Participants emphasized the need for health care systems to prioritize services that are easily accessible and tailored to the unique health challenges faced by older individuals living with HIV. Participants noted that they often had complex, overlapping medical and social needs that required coordinated care across multiple disciplines. While most participants described positive experiences with previous care, they also discussed challenges obtaining information and reaching the appropriate care provider. Several participants specifically recounted situations in which accessing services and support felt very difficult. One participant shared the following:

You can go to a family doc[tor] if you have one and that is so great—again, if you have one. But the issue becomes when the doc refers you to one specialist who refers you to another then back to your family doc and you repeat because no one knows how to care for you.

Interview participant 11; man; White individual; aged 52 years

Therefore, participants described desiring a health care system with the ability to easily access services from care providers.

#### Intersecting Sociocultural and Demographic Factors Complicate Receipt of Care for Older Adults Living With HIV

Participants highlighted that they often faced systemic barriers that hindered their ability to access health care services. These challenges were exacerbated by age-related biases within the system, which was not designed to fully address or accommodate their unique needs. Participants highlighted that those with lower incomes may experience additional obstacles, including lower access to resources; heightened stigma; and greater socioeconomic disadvantages that impact their health, such as the inability to purchase both food and medication. Participants agreed that virtual care has the potential to alleviate some of these systemic barriers by providing more accessible and flexible health care options. However, for virtual care to be an effective solution, it must address the challenges associated with digital literacy and technology access. One participant pointed out the following:

The level that we’re to access the services at, is a bit crazy and some might have challenges. Like this morning, I tried to go on the touch screen, and I can’t get it on to use my phone. And then I’m seeing that there is no Internet service.

Interview participant 5; man; Black individual; aged 56 years

Moreover, to ensure that virtual care is equitable, it is crucial that health care providers are aware of and address the specific needs and preferences of older adults. For instance, participants noted that they preferred a larger screen for virtual consultations, such as a tablet, over smaller devices such as phones.

#### Peer Support Is Beneficial but Should Not Replace Formal Health Care Services and Supports

Support systems among peers were noted as critical in the management of chronic conditions among older people living with HIV. Peer support groups offered participants emotional and practical guidance from individuals who shared similar experiences, fostering a sense of community and reducing feelings of isolation. Peer interactions were also thought to provide valuable insights into managing HIV and navigating the health care system as an older person as they offered lived experiences and practical advice that complemented professional care. One participant noted the positive impact of peer support, stating the following:

I found that connecting with others who are dealing with the same condition really helped me cope better and stay informed about my health.

Interview participant 3; man; South Asian individual; aged 82 years

In the context of peer support, health management and the pursuit of independence emerged as critical themes in the experiences of older adults living with HIV, particularly within a virtual care framework. Participants relied heavily on peer support to navigate the health care system, highlighting the need for virtual care models that offer tailored resources and programs to address their unique needs. One participant emphasized the value of community-based programs, noting the following:

First of all, it’s a safe place for LGBTQ members. And it’s a positive place for me because it’s 50 plus. Basically, it focuses on age and being gay and what more can we want. Canada is great—it won’t discriminate by colour in medical settings, but sometimes we can’t find what we need.

Interview participant 11; man; White individual; aged 52 years

Despite the availability of such programs, challenges remained, particularly in accessing mental health services. The participants wished for more targeted online resources for aging, stress, or depression beyond medication. Furthermore, virtual care could enhance independence in health management by facilitating regular interactions with health care providers through digital platforms. Participants expressed a need for frequent virtual check-ins, such as weekly phone calls, to better monitor and manage their health conditions and maintain a sense of connection and support.

Participants noted that, while peer support was invaluable, they were cautious that their reliance on individuals with lived experience was due to systemic gaps in health care provision and the lack of formal support structures for addressing the complex interplay of health and social circumstances affecting older adults living with HIV. One participant remarked the following:

If I get sick, then I lose my job, and then the chance of losing my housing rise exponentially and so basically I have to have friends who have a house or know someone who is hiring to pay for medicine.

Interview participant 1; woman; White individual; aged 57 years

The same participant shared the following:

If you have money, you’ll get the healthcare in the time that you need it. And if you don’t, well, then good luck.

Another participant shared the following:

When you’re first diagnosed, all you’re focused on is survival. You aren’t trying to find services; you meet people who tell you what services exist. But the healthcare system, the focus isn’t on us, who’ve been around for a long time, and now we need different supports in place than we did 20 years ago. So, we’re stuck with the same services.

Interview participant 2; woman; First Nations individual; aged 57 years

#### Virtual Care Can Address Barriers to Care With Access Caveats

Participants noted that the integration of virtual care has the potential to alleviate some of these challenges by offering more flexible and accessible health care options, thereby significantly reducing the logistical challenges associated with in-person visits, as noted by a participant who appreciated the time savings:

I had a bone density scan...when I had it, he wanted to make an appointment, and I had the choice between virtual or going down to the office. I saved like 4 hours.

Interview participant 12; man; First Nations individual; aged 51 years

Another participant shared the following:

When the place is very cold and you got to catch two bus, three busses to meet your doctor, and pay for it each time, we can do virtual. Then when it’s nice outside whether we don’t mind going and spending the time and go out and see.

Interview participant 10; woman; mixed heritage; aged 54 years

However, one participant wisely pointed out the following:

But it has to be only when minor consultation is required...it should [not] replace the face-to-face consultation.

Interview participant 9; man; White individual; aged 59 years

Participants emphasized the importance of effective communication and continuity of care within the virtual geriatric-HIV care model. They expressed a strong desire for seamless interactions among care providers to prevent disruptions in their treatment and communication. This continuity was described as crucial for maintaining the stability of their HIV management, including regular blood work and medication adjustments. Although participants generally reported positive interactions with health care providers, challenges arose in distinguishing between symptoms related to HIV and those associated with normal aging. One participant remarked the following:

Sometimes it’s hard to tell whether a symptom is due to the HIV or just getting older. Clear communication and understanding from my healthcare provider are essential to figuring out what’s really going on.

Interview participant 9; man; White individual; aged 59 years

To address these complexities, participants recommended that novel care models integrate mechanisms for clear, ongoing communication and coordination among health care providers, ensuring that specialized care is tailored to meet the intricate needs of older adults living with HIV.

### Phase 2: Ideating Solutions

Participants suggested improvements to current care models for older adults living with HIV and proposed their wishes regarding virtual care through a series of activities.

#### Importance of Flexible Care Delivery

Participants highlighted the importance of a multimodal care approach to enhance coordinated care and access to health information for older adults living with HIV. One participant explained the following:

...we need it [virtual care] to be offered in a lot of ways, so not just phones because not everyone has one, not everyone has a cell phone, maybe computer, so offer a lot of ways like a phone, cellphone, tablet, computer.

Phase-2 participant 1; man; White individual; aged 56 years

Participants also noted that the approach should integrate various modes of care delivery, such as in-person consultations, virtual visits, and digital health records, to create a more seamless, continuous, and comprehensive health care experience. One participant shared the following:

...you can’t just have it be one approach, sometimes you need to come in, sometimes I might need a virtual visit, sometimes I might want to send information, we need options.

Phase-2 participant 4; woman; Black individual; aged 71 years

#### Education and Communication Regarding Technology Use and Privacy

The need for clear communication regarding new technologies was a recurring theme among participants. They emphasized that educational demonstrations are essential in helping patients understand and effectively use new technological tools. One participant said the following:

...the worst thing someone can do is if you assume we know what we are doing with technology, so it doesn’t have to be a doctor but some instructions. I sometimes use Zoom but then they say go online and upload, I have no idea how to do that.

Phase-2 participant 6; man; White individual; aged 62 years

Transparency regarding technology use in virtual care emerged as a crucial concern for participants. Participants emphasized the need for clear communication about who accesses patient information, how it is used, and the procedures for withdrawing consent. In the context of virtual care, the digital environment can heighten concerns about privacy and confidentiality, making it imperative to establish transparent practices in data privacy and security. Participants indicated that, in virtual settings, where information is often shared and stored electronically, patients may feel more vulnerable and uncertain about their data. Therefore, ensuring that patients are well informed about the handling of their health information is essential for building trust and fostering a sense of comfort with using virtual care services. One participant explained the following:

...sometimes they think we are old, so we don’t understand, but I know about technology and so I want to know who is going to look at my stuff in order to trust you. If I don’t know, I won’t trust this [virtual care].

Phase-2 participant 8; woman; Indian-Caribbean individual; aged 54 years

Participants also underscored the need for transparency regarding the environment in which virtual care appointments are conducted. They expressed concerns about ensuring that these appointments occur in private and secure settings to protect patient confidentiality. Specifically, participants highlighted the importance of knowing whether providers are conducting virtual appointments from locations that guarantee privacy and maintain the integrity of sensitive health information:

I don’t want the doctor to be like talking about HIV with their friends in the background or at Starbucks.

Phase-2 participant 8; woman; Southeast Asian individual; aged 70 years

#### Reminder Systems

Participants suggested the implementation of reminder systems for appointments and follow-ups to ensure continuity of care and reduce missed appointments. Such systems can play a crucial role in maintaining regular contact between patients and health care providers, thereby improving adherence to treatment plans and enhancing health outcomes. By leveraging technology to provide timely reminders, health care systems can support patients in managing their care more effectively and reduce the risk of lapses in treatment. One participant shared the following:

...maybe it’s age, maybe it’s life, but anything to make it easier is good. Remind me when to log in, when to book an appointment, what to do, who to see, even the name, just anything to make life easier when it comes to doctors because sometimes, I see so many and now maybe a geriatrician.

Phase-2 participant 4; woman; Black individual; aged 71 years

### Phase 3: Designing the Action Plan

#### Overview

Phase 3 entailed participants co-designing and planning the necessary actions and initiatives to achieve the desired future for virtual geriatric-HIV care as outlined in phase 2. Participants concluded phase 3 by refining the ideas and virtual geriatric care models co-designed in phase 2 and phase 3. This involved prioritizing linguistic and cultural equity.

#### Linguistic and Cultural Equity

Participants highlighted the need for the virtual geriatric care model to incorporate linguistic and cultural considerations to ensure equitable care for the population of older adults living with HIV. Participants shared stories exemplifying the diversity of the geriatric-HIV community (inclusive of several underserved populations) and emphasized the importance of increasing services to these populations. One participant stated the following:

...not everyone speaks English, especially when they come here and so we need to give back and help them. It’s like if you dropped someone in their countries, it’s like worse than a maze.

Phase-3 participant 11; man; White individual; aged 69 years

Participants noted that there needs to be health- and technology-related information in multiple languages and that service providers should make translation services known to their patients. Participants also suggested that health care providers offer education that addresses many of the culturally related myths and concerns of individuals about aging with HIV. One participant said the following:

...doctors and nurses need to look at who they are serving that is different than them. Not everyone understands HIV is not a life sentence so tell certain people that. Or maybe someone eats food that is different, not everyone understands quinoa. Maybe recommend cultural food.

Phase-3 participant 1; woman; Black individual; aged 51 years

#### Consider Affordability

Participants were broadly aware of the fact that the cost of technology could be a barrier to many older adults living with HIV. Participants highly recommended that geriatric-HIV virtual care models be made more accessible by offering subsidized internet access or technological devices. Participants also shared that some patients may have never used technology before virtual care due to high costs and, therefore, may need training on how to best use the technology for care. Participants recognized that health care providers may not have the capacity to teach their clients, and as such, someone else in the health care organization should. For instance, participants suggested that perhaps calling a patient on a landline or inviting the patient to in-person training could help support their use of virtual modalities for health care in the future. One participant described the following:

...maybe invite someone to a place in person just even once or call them and walk them through something like how to turn their video on or how to send a text to a doctor.

Phase-3 participant 17; woman; Black individual; aged 54 years

Participants emphasized the importance of early education on technology, as previously underscored in the other phases of the study.

Participants expressed significant concern about the high cost of HIV-related medications, particularly for those aged <65 years, who are ineligible for government-funded drug coverage in Ontario. Some participants felt that health care providers were not advocating enough for the use of lower-cost alternatives such as generic medications, which could be just as effective as brand-name drugs. For instance, one patient mentioned the following:

It [medication] can be the choice between food, rent or medications at times, so I want someone who maybe says a cheaper supplement or maybe what medication I can maybe take a different type of. Anything to keep the cost low.

Phase-3 participant 9; woman; Asian individual; aged 70 years

Participants indicated that a more proactive stance from providers in promoting cost-effective treatment options, both for HIV and age-related conditions, would improve their overall care experience.

#### Person-Centered Care for Age-Related Health Issues

Participants highlighted the importance of having health care providers who understood the dual challenges of aging and living with HIV. They emphasized that a key feature of an effective geriatric virtual care program would be the integration of expertise in both areas. This holistic approach was seen as essential for managing the complexities of age-related conditions alongside HIV, with many noting that current segmented specialist care often leads to fragmented treatment, polypharmacy, and inconsistent care coordination. An integrated model was viewed as a potential solution to improve care continuity and reduce these challenges.

Participants emphasized the importance of incorporating cognitive health screening and related support services into the virtual care model. This integration would address current gaps in HIV care by ensuring that the program not only focuses on medical treatment but also considers the emotional aspects of aging with HIV. Current gaps within HIV care were re-emphasized, and participants noted that, for uptake of the geriatric-HIV virtual care model to occur, participants needed to “feel as if the doctor or care professionals care of us as a person, not just a billable code or something, but to really understand how much [older adults living with HIV] struggle” (phase-3 participant 8; man; White individual; aged 67 years)*.*

#### Patient Resilience and Support

Participants emphasized a virtual geriatric-HIV model of care that incorporates support networks to enhance patient resilience to living with HIV in older age. The involvement of trusted individuals such as existing care providers, friends, or service agencies was deemed essential to care by participants. This holistic support system helps patients navigate the challenges of their health journey, promotes a sense of connection and belonging, and strengthens their overall well-being. By embedding these peer support networks within the virtual care model, the program can provide a more robust and empathetic approach to patient care. One participant described the following:

I’d trust anything that [Community Agency] recommended, so if you bring your services to them so that they encourage them, I’ll trust that it would help me.

Phase-3 participant 4; woman; White individual; aged 58 years

Moreover, participants emphasized that a virtual geriatric-HIV model of care should include features such as virtual support groups, peer mentoring programs, and online forums where individuals with similar experiences can connect and share insights. In addition, incorporating the peer mentorship system could allow patients to receive guidance and encouragement from those who have successfully managed similar health conditions.

## Discussion

### Principal Findings

This study was conducted to co-design a geriatric-HIV virtual care model for older adults living with HIV. The lack of tailored interventions that consider this population’s specific needs and preferences necessitated a co-design approach. Using EBCD methodology [84,85,92], we engaged older adults living with HIV to collaboratively codevelop a virtual care model. Through interviews, focus groups, and a co-design workshop, participants identified key needs such as robust support systems, health management resources, and effective communication strategies. They cocreated and refined solutions including multimodal care options, technology transparency recommendations, and reminder systems.

Using co-design methodologies ensured that the voices and experiences of older adults living with HIV were central to the development and evaluation of the virtual care models. This inclusive, user-centered approach fosters solutions that are more likely to be accepted and effective as they are directly informed by the end users’ needs and preferences. Engaging participants in the research process empowers them and enhances the relevance and applicability of the findings. A strength of this study was the inclusion of many English-as-a-second-language individuals and immigrants to Canada, who are typically underrepresented in research [109]. This diverse participant pool enhanced the transferability of the findings, ensuring that the developed virtual care models were relevant and accessible to a broader range of older adults living with HIV. By actively including these often-excluded groups, this study addresses potential disparities in health care access and provides insights into the unique needs and challenges faced by these populations. Thus, this research provides practical insights into the real-world application and potential barriers to adoption of virtual care models. This dual focus ensures that this study not only explores the theoretical potential of the interventions but also addresses the pragmatic challenges that might arise during implementation. As a result, the findings are highly relevant for informing policy and practice, ultimately contributing to the advancement of care for older adults living with HIV.

The findings presented in this paper have been echoed in various other studies with other populations. Research among rural and urban adults has shown high satisfaction with virtual care [110]. In addition, studies on virtual care for substance use indicate its ability to bridge service gaps, particularly in rural areas, reinforcing the importance of multimodal care options and effective communication [111]. Canadian case studies further demonstrate the necessity of involving users in the development of virtual care tools to ensure their effectiveness and transparency [112].

Our findings underscore the importance of integrating cultural and linguistic equity into virtual care models, which has substantial implications for enhancing health care accessibility and outcomes for older adults living with HIV. Research has shown that sociocultural stigma can severely impact health care engagement and access [36,113], highlighting the importance of designing solutions that are sensitive to these issues.

Involving older adults living with HIV in co-designing care solutions is essential to ensure that their needs are met [90]. Engaging older adults living with HIV in the development process ensures that the solutions are tailored to their specific needs and the challenges they face, thus enhancing their effectiveness and acceptance [90]. This study’s findings have important implications for health policy, particularly regarding the integration of virtual care models for older persons living with HIV. Existing reviews have found that virtual geriatric-HIV programs are limited [17] despite their proposed benefits. Policy makers should consider the advantages of virtual care in addressing the unique needs of this population, especially in contexts in which traditional health care access is limited. Our study highlights that participants perceive virtual care to be feasible and acceptable for meeting their geriatric-HIV care needs, suggesting that policies should support the expansion of telehealth services and funding for technological infrastructure. In addition, policies should focus on inclusivity by ensuring that virtual care models are accessible to non-English speakers and diverse cultural groups who may have differing views on HIV care [50,114]. This could involve implementing language support services and cultural competency training for health care providers to enhance the effectiveness of virtual interventions. This could also involve funding geriatric health care settings such that they can hire multilingual staff, partner with translation services, and provide localized materials.

In practice, this study underscores the importance of adopting virtual care models as a viable alternative or complement to traditional in-person geriatric consultations for older persons living with HIV [67]. Health care providers should be encouraged to incorporate these models into their service offerings, with attention to user feedback and the co-design process highlighted in this study. Research emphasizes the need for health care professionals to collaborate with patients in co-design methodologies [115] to enhance patient participation in health care services. Projects involving creative workshops have shown that health care professionals can gain valuable insights and training through active participation in co-design processes [116]. In addition, discussions on EBCD underscore the necessity of equipping health care professionals with the tools and knowledge to effectively engage in patient-centered design and improvement initiatives [117]. Overall, training health care providers in these collaborative approaches is essential for fostering meaningful partnerships with patients in health care settings. For example, health care settings could employ staff trained in the use of virtual platforms and equipped with resources to support patients in navigating these technologies. Optimal methods for training older adults to use technology involve a combination of hands-on practice, clear instructions, and personalized support tailored to individual needs and cognitive abilities [118]. Effective approaches often include guided action and attention training, addressing fears, building confidence, and providing ongoing support, with a focus on practical, relevant applications that motivate learners [118,119]. Moreover, the integration of language support and culturally sensitive practices is crucial to ensure that virtual care is accessible to diverse populations. Other studies of virtual care have found that features such as multilingual support, screen readers, and voice commands can make virtual care more accessible to diverse patient populations [120]. Moreover, integrating peers into virtual care models can be achieved through comprehensive support, training, and collaboration across various providers [121].

To test the virtual geriatric care model, future research should use randomized controlled trials comparing virtual geriatric-HIV care with traditional care in terms of patient satisfaction, health outcomes, and cost-effectiveness. Specific technological platforms and features should also be evaluated to determine their effectiveness in addressing the unique needs of culturally diverse older adults living with HIV. In addition, implementation studies should examine barriers and facilitators in various care settings to optimize and scale the model effectively. Future research should build on the findings of this study by exploring the long-term impacts of virtual care models on health outcomes and quality of life for older persons living with HIV. Longitudinal studies could provide insights into the sustainability and efficacy of these interventions over time. In addition, research should investigate the effectiveness of different technological platforms and features in meeting the needs of culturally diverse older persons living with HIV. Existing comparative studies have not assessed virtual care against traditional models in the context of HIV. In addition, issues of equity and access must be addressed to avoid further disadvantaging vulnerable individuals who may lack access to necessary technology or resources. Furthermore, exploring the barriers to and facilitators of implementation in various settings, such as acute and community care, can offer valuable information for refining and scaling virtual geriatric-HIV care models. Finally, future research should continue to address the needs of non-English speakers and other underserved groups, ensuring that virtual care remains inclusive and equitable.

### Limitations

This study, while innovative in its co-design approach to developing a virtual care model for older adults living with HIV, has noted limitations. First, the sample size was relatively small and geographically limited to a dense urban center, which may impact the transferability of the findings. Most participants were already engaged with technology, which may not accurately represent the broader population of older adults living with HIV who have varying levels of technological proficiency. This existing familiarity could have positively biased the feedback on virtual care models as these participants, who were actively seeking health care services, might be more comfortable and adept with digital tools. This may not be representative of others who avoid health care and may struggle more with virtual care platforms. Consequently, the findings may overestimate the acceptability of such interventions for the entire demographic, potentially overlooking significant barriers experienced by those less familiar or comfortable with technology. Future research should aim to include a more diverse sample in terms of technological experience to ensure that the developed care models are inclusive and accessible to all older adults living with HIV. Finally, geriatric-HIV care providers and community agencies were not engaged as formal participants in the co-design phases. Their exclusion may have limited insights into the clinical and service delivery perspectives necessary for developing a more holistic and integrated care model. Engaging these stakeholders could have addressed gaps related to continuity of care and support services. Future iterations of this model would benefit from the involvement of health care providers and community organizations to ensure that the virtual care system is aligned with existing services and adequately meets the needs of older adults living with HIV.

### Conclusions

This study highlights the crucial need for socioculturally appropriate virtual care models for older persons living with HIV. Using an EBCD approach, we engaged older persons living with HIV in co-designing a virtual care model that addresses their unique needs and challenges. The findings reveal several key areas for improvement in virtual geriatric-HIV care, including the necessity for robust support systems, effective communication strategies, and tailored health management resources. Participants identified critical needs such as the integration of multimodal care options, transparency in technology use, and the implementation of reminder systems to enhance patient engagement and continuity of care. They also emphasized the importance of linguistic and cultural equity, recommending that virtual care models incorporate diverse language options and cultural considerations to ensure accessibility and relevance. In addition, affordability was highlighted as a significant barrier, with participants advocating for subsidized technology and training to overcome economic challenges. Policy makers should consider the advantages of virtual care for older persons living with HIV and support the expansion of telehealth services and technological infrastructure. Future research should explore the long-term impacts of virtual care on health outcomes and quality of life on older persons living with HIV as well as evaluating different technological platforms and features. By embracing the diverse voices of older persons living with HIV, this study paves the way for a more inclusive, innovative, and equitable virtual future of care.

## Appendix

Multimedia Appendix 1 Demographic information for participants in phases 1, 2, and 3 (N=19).

## Abbreviations

CBPR: community-based participatory research
EBCD: experience-based co-design
