# Supplementary material for: Socioculturally Appropriate Internet-Based Geriatric Care Model for Older Adults Living With HIV: Experience-Based Co-Design Approach
Source: JMIR Aging. 2025 May 27;8:e67122. doi: 10.2196/67122 (PMC12152441; doi:10.2196/67122)
Supplement: Multimedia Appendix 1 [file aging_v8i1e67122_app1.docx]

**Multimedia Appendix 1.** Demographic information for participants in phases 1, 2, and 3 (N=19).

| Demographic variable and response options | | Phase 1 (n=14), n (%) | Phase 2 (n=10), n (%) | Phase 3 (n=12), n (%) |
| --- | --- | --- | --- | --- |
| **Age group (y)** | | | | |
|  | 50-54 | 4 (29) | 2 (20) | 2 (17) |
|  | 55-59 | 4 (29) | 3 (30) | 3 (25) |
|  | 60-64 | 0 (0) | 1 (10) | 1 (8) |
|  | 65-69 | 3 (21) | 1 (10) | 3 (25) |
|  | 70-74 | 0 (0) | 3 (30) | 3 (25) |
|  | 75-79 | 1 (7) | 0 (0) | 0 (0) |
|  | ≥80 | 2 (14) | 0 (0) | 0 (0) |
| **Gender** | | | | |
|  | Man | 10 (71) | 6 (60) | 7 (58) |
|  | Woman | 4 (29) | 3 (30) | 3 (25) |
|  | Nonbinary | 0 (0) | 0 (0) | 1 (8) |
|  | Two spirit | 0 (0) | 1 (10) | 0 (0) |
|  | Transgender | 0 (0) | 0 (0) | 1 (8) |
| **Living setting** | | | | |
|  | Urban | 9 (64) | 8 (80) | 9 (75) |
|  | Suburban | 2 (14) | 1 (10) | 1 (8) |
|  | Rural | 3 (21) | 1 (10) | 1 (8) |
|  | No response | 0 (0) | 0 (0) | 1 (8) |
| **Country of birth** | | | | |
|  | Canada | 6 (43) | 4 (40) | 4 (33) |
|  | Other | 8 (57) | 5 (50) | 6 (50) |
|  | Prefer not to say | 0 (0) | 1 (10) | 1 (8) |
|  | No response | 0 (0) | 0 (0) | 1 (8) |
| **Length of time residing in Canada (y)** | | | | |
|  | 0-5 | 4 (29) | 1 (10) | 2 (17) |
|  | 6-10 | 1 (7) | 1 (10) | 1 (8) |
|  | >10 | 9 (64) | 2 (20) | 2 (17) |
|  | Whole life | 0 (0) | 3 (30) | 3 (25) |
|  | Prefer not to say | 0 (0) | 2 (20) | 2 (17) |
|  | No response | 0 (0) | 0 (0) | 2 (17) |
| **English as first language** | | | | |
|  | Yes | 8 (57) | 4 (40) | 6 (50) |
|  | No | 6 (43) | 6 (60) | 6 (50) |
| **Race or ethnicity** | | | | |
|  | Asian—South (eg, Indian, Pakistani, and Sri Lankan) | 3 (21) | 1 (10) | 1 (8) |
|  | Asian—Southeast (eg, Malaysian, Filipino, and Vietnamese) | 0 (0) | 1 (10) | 1 (8) |
|  | Black—African (eg, Ghanaian, Kenyan, and Somali) | 1 (7) | 0 (0) | 0 (0) |
|  | Indian—Caribbean (eg, Guyanese with origins in India) | 1 (7) | 3 (30) | 1 (8) |
|  | Indigenous | 2 (14) | 3 (30) | 3 (25) |
|  | Latin American (eg, Argentinean, Chilean, and Salvadoran) | 0 (0) | 2 (20) | 4 (33) |
|  | Middle Eastern (eg, Egyptian, Iranian, and Lebanese) | 0 (0) | 0 (0) | 2 (17) |
|  | Mixed heritage (eg, Black-African and White—North American) | 1 (7) | 0 (0) | 0 (0) |
|  | White—European (eg, English, Italian, and Portuguese) | 2 (14) | 0 (0) | 0 (0) |
|  | White—North American (eg, Canadian and American) | 4 (29) | 0 (0) | 0 (0) |
| **Sexual orientation** | | | | |
|  | Heterosexual | 5 (36) | 2 (20) | 2 (17) |
|  | Bisexual | 0 (0) | 2 (20) | 0 (0) |
|  | Gay | 7 (50) | 6 (60) | 3 (25) |
|  | Lesbian | 0 (0) | 0 (0) | 7 (58) |
|  | Queer | 0 (0) | 0 (0) | 0 (0) |
|  | Two spirit | 0 (0) | 1 (10) | 1 (8) |
|  | Asexual | 2 (14) | 0 (0) | 0 (0) |
| **Household income before taxes** | | | | |
|  | CAD $0-$29,999 (US $0-$21,644.30) | 9 (64) | 8 (80) | 9 (75) |
|  | CAD $30,000-$59,999 (US $21,645.10-$43,289.40) | 0 (0) | 1 (10) | 1 (8) |
|  | CAD $60,000-$89,999 (US $43,290.10-$64,934.50) | 3 (21) | 1 (10) | 1 (8) |
|  | CAD $90,000-$119,999 (US $64,935.20-$86,579.50) | 1 (7) | 0 (0) | 0 (0) |
|  | CAD $120,000-$149,999 (US $86,580.20-$108,225) | 0 (0) | 0 (0) | 0 (0) |
|  | ≥CAD $150,000 (US $108,225) | 1 (7) | 0 (0) | 1 (8) |
| **Number of people in the household** | | | | |
|  | 0 (eg, precariously housed) | 1 (7) | 0 (0) | 0 (0) |
|  | 1 | 8 (57) | 8 (80) | 9 (75) |
|  | 2 | 5 (36) | 2 (20) | 3 (25) |
| **Religion or spiritual affiliation** | | | | |
|  | Agnosticism (agnostic) | 1 (7) | 2 (20) | 2 (17) |
|  | Atheism (atheist) | 1 (7) | 0 (0) | 0 (0) |
|  | Buddhism (Buddhist) | 0 (0) | 1 (10) | 1 (8) |
|  | Christianity (Christian) | 7 (50) | 3 (30) | 4 (33) |
|  | Hinduism (Hindu) | 1 (7) | 0 (0) | 0 (0) |
|  | Indigenous spirituality | 1 (7) | 0 (0) | 0 (0) |
|  | Islam (Muslim) | 1 (7) | 0 (0) | 0 (0) |
|  | Judaism (Jewish) | 0 (0) | 1 (10) | 1 (8) |
|  | Sikhism (Sikh) | 0 (0) | 0 (0) | 0 (0) |
|  | Spiritual | 2 (14) | 1 (10) | 1 (8) |
|  | No religion | 0 (0) | 2 (20) | 3 (25) |
|  | More than one faith or religion | 0 (0) | 0 (0) | 0 (0) |
| **Level of education** | | | | |
|  | Lower than high school | 3 (21) | 2 (20) | 3 (25) |
|  | High school or equivalent | 2 (14) | 0 (0) | 0 (0) |
|  | Some college or university | 3 (21) | 5 (50) | 5 (42) |
|  | Degree or diploma from college or university | 3 (21) | 1 (10) | 1 (8) |
|  | Graduate or professional degree | 3 (21) | 2 (20) | 3 (25) |
| **Disabilities^a^** | | | | |
|  | Sensory impairment—vision | 5 (36) | 1 (10) | 1 (8) |
|  | Sensory impairment—hearing | 1 (7) | 2 (20) | 2 (17) |
|  | Mobility impairment (eg, use of a cane) | 2 (14) | 2 (20) | 3 (25) |
|  | Learning disability (eg, ADHD^b^ or dyslexia) | 0 (0) | 3 (30) | 4 (33) |
|  | Mental health disorder | 3 (21) | 2 (20) | 2 (17) |
|  | Disability or impairment not listed | 0 (0) | 4 (40) | 5 (42) |
|  | No disability or impairment | 3 (21) | 0 (0) | 0 (0) |
|  | No response | 4 (29) | 0 (0) | 0 (0) |
| **Comorbidities^c^** | | | | |
|  | Diabetes | 0 (0) | 2 (20) | 2 (17) |
|  | High blood pressure | 3 (21) | 3 (30) | 4 (33) |
|  | High cholesterol | 3 (21) | 4 (40) | 4 (33) |
|  | Heart disease | 0 (0) | 1 (10) | 1 (8) |
|  | Cancer | 2 (14) | 1 (10) | 3 (25) |
|  | COPD^d^ | 1 (7) | 2 (20) | 2 (17) |
|  | Asthma | 1 (7) | 2 (20) | 2 (17) |
|  | Arthritis | 2 (14) | 2 (20) | 2 (17) |
|  | Dementia | 1 (7) | 2 (20) | 2 (17) |
|  | Chronic pain | 3 (21) | 3 (30) | 4 (33) |
|  | Diagnosed mental health condition | 3 (21) | 4 (40) | 4 (33) |
|  | Other | 1 (7) | 1 (10) | 1 (8) |
|  | No response | 4 (29) | 0 (0) | 0 (0) |
| **Access to a computer** | | | | |
|  | Yes | 9 (64) | 8 (80) | 10 (83) |
|  | No | 1 (7) | 2 (20) | 2 (17) |
|  | No response | 4 (29) | 0 (0) | 0 (0) |
| **Access to a smartphone** | | | | |
|  | Yes | 9 (64) | 7 (70) | 9 (75) |
|  | No | 1 (7) | 3 (30) | 3 (25) |
|  | No response | 4 (29) | 0 (0) | 0 (0) |
| **Access to internet connectivity** | | | | |
|  | Yes | 9 (64) | 7 (70) | 9 (75) |
|  | No | 1 (7) | 3 (30) | 3 (25) |
|  | No response | 4 (29) | 0 (0) | 0 (0) |
| **Required assistance with internet use** | | | | |
|  | Yes | 3 (21) | 2 (20) | 3 (25) |
|  | No | 7 (50) | 7 (70) | 8 (67) |
|  | No response | 4 (29) | 0 (0) | 0 (0) |
|  | Prefer not to say | 0 (0) | 1 (10) | 1 (8) |

^a^Some participants selected more than one disability.

^b^ADHD: attention-deficit/hyperactivity disorder.

^c^Some participants selected more than one comorbidity.

^d^COPD: chronic obstructive pulmonary disease.
